# The expression of mismatched repair genes and their correlation with clinicopathological parameters and response to neo-adjuvant chemotherapy in breast cancer

**DOI:** 10.1186/1477-7800-4-5

**Published:** 2007-02-14

**Authors:** Chintamani, Binita P Jha, Vimal Bhandari, Anju Bansal, Sunita Saxena, Dinesh Bhatnagar

**Affiliations:** 1 Vardhman Mahavir Medical College Safdarjang Hospital New Delhi, India; 2 Department of surgery, Vardhman Mahvir Medical College, New Delhi, India; 3 Institute of Pathology, Indian Council Of Medical Research, New Delhi, India

## Abstract

**Background:**

The DNA mismatch repair (MMR) pathway is an important post-replicative repair process. It is involved in the maintenance of genomic stability and MMR genes have therefore been named the *proofreaders *of replicating DNA. These genes repair the replicative errors of DNA and are thus imperative for genomic stability.

The MMR genes have been found to be involved in promoting cytotoxicity, apoptosis, p53 phosphorylation and cell cycle arrest following exposure to exogenous DNA damaging agents. Loss of MMR function prevents the correction of replicative errors leading to instability of the genome, and can be detected by polymorphisms in micro satellites (1–6 nucleotide repeat sequences scattered in whole of the genome). This phenomenon, known as micro satellite instability (MSI), is a hallmark of MMR dysfunction and can be used as a marker of MMR dysfunction in colorectal and other malignancies. An alternative method for detection of MMR dysfunction is to test the expression of protein products of the MMR genes by immunohistochemistry (IHC), as mutations in these genes lead to reduced or absent expression of their gene products.

Correlation between loss of MMR function and clinical, histopathological, behavioral parameters of the tumor and its response to chemotherapy in breast cancers may be of value in predicting tumor behavior and response to neoadjuvant chemotherapy (NACT). Neoadjuvant chemotherapy is an integral part of multimodal therapy for locally advanced breast cancer and predicting response may help in tailoring regimens in patients for optimum response.

**Materials:**

After approval by the IRB(Institutional Review Board) and ethical committee of the hospital, 31 cases of locally advanced breast carcinoma (LABC) were studied to assess the correlation between MMR dysfunction, clinicopathological parameters and objective clinical response to neoadjuvant chemotherapy using immunohistochemistry. The immunohistochemical analysis for four MMR protein products -MLH1, MSH2, MSH6 and PMS2 was done in the pre NACT trucut biopsy specimen and after three cycles of NACT with C AF (cyclophosphamide, adriamycin, 5-fluorouracil) regimen, in the modified radical mastectomy specimen.

**Results and conclusion:**

There was no significant correlation observed between expression of MMR proteins and age, family history, tumor size or histological type. However there was a statistically significant negative correlation between MLH1, MSH2 expression and histological grade. There was also a negative correlation observed between PMS2 expression after neo-adjuvant chemotherapy and clinical response. Cases with high post NACT expression of PMS2 were poor responders to chemotherapy. MSH6 was the most frequently altered MMR gene, with a negativity rate of 48% and the patients with high expression responded poorly to NACT. The study highlights the possible role of MMR expression in predicting aggressive tumor behavior (histological grade) and response to neoadjuvant chemotherapy in patients with LABC.

## Background

Breast cancer is second only to cancer of the cervix in India[1,2].The standard of care in India for locally advanced breast cancer is neoadjuvant chemotherapy(NACT) followed by surgery in the form of modified radical mastectomy and subsequently three more cycles of adjuvant chemotherapy. NACT facilitates local as well as distant control of the disease and provides an *in vivo *chemosensitivity test for a particular regime. It is vital to predict response to chemotherapy in order to tailor the regime in a particular patient for an optimum response and to avoid chemotoxicity in a non-responder. Various markers like p-glycoprotein, p53, apoptotic markers and toxicity etc. have been studied to assess and predict response to NACT but the search for an ideal predictor is still on[3,4].

Mismatch repair(MMR) genes first came into focus due to their role in hereditary nonpolyposis colon cancer (HNPCC) syndrome [5-10]. The dysfunction of these genes has also been implicated in sporadic colorectal, gastric, endometrial, prostatic, and hematological malignancies. These genes repair the replicative errors of DNA and are imperative for stability of the genome. MMR proteins have been found to be involved in promoting cytotoxicity, apoptosis and p53 phosphorylation. They have also been found to be responsible for cell-cycle arrest following exposure to exogenous DNA damaging agents, including those agents that form DNA adducts not removed by MMR, like thioguanine, cisplatin, carboplatin, doxorubicin and etoposide [7-10].

With the loss of MMR function replicative errors are not corrected, leading to instability of the genome. This can be detected by polymorphisms in micro satellites (1–6 nucleotide repeat sequences scattered in whole of the genome) [7-10]. This phenomenon, known as micro satellite instability (MSI) is a hallmark of MMR dysfunction in colorectal and other malignancies. An alternative method for detection of loss of MMR function is to test the expression of protein products of the MMR genes i.e. MLH1, MSH2, MSH6 and PMS2 by immunohistochemistry (IHC), as mutations in these genes lead to reduced or absent expression of their gene products [7-10].

In colorectal cancers, MSI positivity imparts distinct clinicopathological features and response to chemotherapy has been found to be favorable in MSI positive tumors. Thus detection of MMR gene dysfunction is becoming an essential tool in the management of these cancers. In breast cancer, the role of MMR genes has been studied increasingly in the past two decades and the detection rate of MSI has been found to vary greatly(5 to 30 %) between various studies [10-13]. There may be a correlation between loss of MMR function in breast cancer and clinical, histopathological, behavioral parameters of the tumor and also its response to chemotherapy [10-14].

We studied 31 cases of locally advanced breast carcinoma (LABC) for correlation between MMR dysfunction, clinicopathological parameters and response to chemotherapy using immunohistochemistry for four MMR protein products -MLH1, MSH2, MSH6 and PMS2, before and after NACT.

## Patients and methods

After approval by the Institutional Review Board and the ethical committee of the hospital, 31 fine needle aspiration cytology (FNAC) proven cases of LABC according to AJCC (American Joint Committee on Cancer) classification were included in the study. The tumor size and the axillary lymph node status were assessed clinically and by using ultrasonography. Core biopsy was performed for immunohistochemical estimations of MMR proteins in the specimen before initiating the chemotherapy. Blood tests, chest radiographs, electrocardiography (ECG) (echocardiography when ECG had a positive finding), liver function tests, bone scan, ultrasonography (USG) of the abdomen, renal function tests were routinely performed in all the cases.

Three cycles of FAC regime (cyclophosphamide 500 mg/m^2^, adriamycin 50 mg/m^2^, 5-fluorourail 500 mg/m^2^) were given at three weekly intervals and the patients were assessed both clinically and by ultrasound for response in the form of reduction in breast tumor size and axillary lymph node status. Patey's modified radical mastectomy was performed three weeks after the last cycle and the mastectomy specimen was examined for pathological response, resected margins, axillary lymph node status and IHC for MMR proteins.

The pathological tumor response was evaluated by size measurement at the time of tumor resection macroscopically and by detecting tumor cell existence (or not) microscopically. Clinical responders were defined as patients with a complete (CR) or partial response (PR) [CR: complete resolution of tumor, PR>50% regression in maximum diameter of initial tumor] after 3 cycles of NACT. Non-responders were patients with a minimal response (MR<50% regression in maximum diameter of initial tumor), no change (NC) or local progression. Pathological complete response (pCR) was defined as absence of any gross or microscopic evidence of residual tumor in the mastectomy specimen i.e. absence of residual invasive or in situ disease following neoadjuvant chemotherapy. Its assessment was done, irrespective of the clinical response status. Clinical response was taken in to consideration for statistical analysis as the pCR was observed in only 2 patients (n = 31).

### Immunohistochemical method

The monoclonal mouse antibody, clone 124 (DAICD) was used. Heating in water broth at 95 degree Celsius for 2 hours in 0.1 M-utrate buffer (6.0 pH) was done for antigen-retrieval. The antibody localization was seen in the cytoplasm of the cells and IHC staining was done by Avidin Biotin technique.

The staining was analyzed as per the intensity and the percentage of positive cells.

H-score was calculated for each of the four MMR proteins and for this purpose the intensity of staining of tissue was scored as 0–3 (**absent, mild, moderate, intense**) and percentage of positive staining cells were scored as 0–4. The H-Score was calculated by multiplying these two scores thus the minimum H-score being 0 and maximum 12.

## Results

31 patients with locally advanced breast cancer were included in this study, the mean age at presentation was 47.3 years,19(61.2%) were postmenopausal and 12 patients(38.7%) were premenopausal. The mean tumor size was 6.7 cms and 22 out of 31 cases (70.9%) were N1 at presentation. Majority of tumors were infiltrating ductal carcinomas (IDC) i.e. 25 out of 31. However two cases of infiltrating lobular carcinomas mixed with IDC and two cases of mucinous carcinoma including one of pure lobular variety were also found (table 1). More than 50% tumors were moderately differentiated while16.70% were poorly differentiated and 30% were well differentiated (table 2).

**Table 1:** 

**Distribution of patients based on the histological types **(n = 31)		
Infiltrating duct carcinoma(IDC)	Infiltrating lobular carcinoma mixed with IDC	Mucinous carcinoma
25 (81.4%)	3 (9.3%)	3 (9.3%)

**Distribution based on the differentiation **(n = 31)		

Well differentiated	Moderately differentiated	Poorly differentiated
9(30%)	17(53.3%)	5(16.7%)

**Table 2:** 

**Mean tumor size before and after NACT**		Mean	N	Std. Deviation	Std. Error Mean
Pair 1	Pre NACT Tumor Size	6.82	31	2.64	.47
	Post NACT Tumor size	4.37	31	2.6171	.4701

**Mean N Stage before and after NACT**					

----------		Mean	N	Std. Deviation	Std. Error Mean
Pair 2	Pre NACT N stage	1.16	31	.52	.13
	Post NACT N stage	.81	31	.65	.12

The objective clinical response rate observed in our study was 54.83% with 17 patients (54.83%) being responders including 2 patients with pathological complete response(table 2). With the confidence limit of 99% paired- t test was applied to the above table and response to NACT in terms of down staging of the tumor size and axillary lymph node status was found to be statistically significant with a P-value of 0.000.The correlation between these two parameters was studied using Spearman's rank correlation coefficient test with a confidence limit of 95%.

### Immunohistochemical analysis

Immunohistochemical results were analyzed using the H score as described abov (tables 3, 4, 5, 6, 7, 8, 9, 10)

**Table 3:** DISTRIBUTION ACCORDING TO H-SCORE MLH1

	Frequency	Percent	Valid percent	Cumulative percent
0	13	41.93	41.93	41.93
2	2	6.45	6.45	48.38
4	2	6.45	6.45	54.83
6	9	29.03	29.03	83.86
9	5	16.7	16.7	100
12	0	0	0	100
Total	31	100	100	

13 cases out of 31 (41.93%) had negative staining (score = 0) for MLH1.Out of the rest 18 cases only 2 had a H-score less than 2 signifying that staining, when present is remarkable.

**Table 4:** MSH 2

	Frequency	Percent	Valid percent	Cumulative percent
0	12	38.71	38.71	38.71
2	8	25.81	25.81	64.52
4	3	9.67	9.67	74.19
6	7	22.58	22.58	96.77
9	1	3.22	3.22	100
12	0	0	0	100
Total	31	100	100	

12 cases out of 31 (38.73%) had negative staining (score = 0) for MSH2.Out of the rest 19 cases the H-score of <2 was found in 8 cases thus the staining for MSH2 was less consistent than MLH1.

**Table 5:** MSH6

	Frequency	Percent	Valid percent	Cumulative percent
0	15	48.39	48.39	48.39
2	5	16.12	16.12	64.57
3	2	6.45	6.45	70.97
4	5	16.12	16.12	87.08
6	1	3.22	3.22	90.3
9	1	3.22	3.22	93.52
12	2	6.45	6.45	100
Total	31	100	100	

15 cases had negative expression for MSH6 and the rest 16 stained variably for MSH6 with some tumors showing H-score of 12.

**Table 6:** PMS2

	Frequency	Percent	Valid percent	Cumulative percent
0	9	29.03	29.03	29.03
2	5	16.12	16.12	45.15
3	2	6.45	6.45	51.60
4	1	3.22	3.22	54.82
6	9	29.03	29.03	83.85
9	2	6.45	6.45	90.3
12	3	9.67	9.67	100
Total	31	100	100	

Amongst all MMR products the H-score was highest forPMS2. Also negativity rate was only 29.03%

**Table 7:** MLH1

SCORE	Percentage of cells staining	Frequency	Percent	Valid percent	Cumulative percent
0	0%	13	41.93	41.93	41.93
1	<5%	0	0	0	41.93
2	5–20%	4	12.90	12.90	54.83
3	20–80%	14	45.16	45.16	100
4	80–100%	0	0	0	

Of the 18 cases staining positive 14 had staining in more than 80 % of cells. This result correlated well with the results of H-scoring.

**Table 8:** MSH2

SCORE	Percentage of cells staining	Frequency	Percent	Valid percent	Cumulative percent
0	0%	12	38.70	38.70	38.70
1	<5%	0	0	0	38.70
2	5–20%	11	35.48	35.48	74.18
3	20–80%	8	25.80	25.80	100
4	80–100%	0	0	0	100

Like the results of H-score for MSH2, score based on percentage of positively staining cells for MSH2 was also low for most of the tumors. 23 cases out of 31 had a score of less than 2.

**Table 9:** MSH6

SCORE	Percentage of cells staining	Frequency	Percent	Valid percent	Cumulative percent
0	0%	15	48.38	48.38	48.38
1	<5%	0	0	0	0
2	5–20%	5	16.12	16.12	64.5
3	20–80%	8	25.80	25.80	90.3
4	80–100%	3	9.67	9.67	100

The results of score based on percentage of positively staining cells were similar to that of H-score with both showing variable staining in positively staining tumors.

**Table 10:** PMS2

SCORE	Percentage of cells staining	Frequency	Percent	Valid percent	Cumulative percent
0	0%	9	29.03	29.03	29.03
1	<5%	0	0	0	29.03
2	5–20%	6	19.35	19.35	48.38
3	20–80%	14	45.16	45.16	93.81
4	80–100%	2	6.45	6.45	100

Only 9 cases had a score of 0 for PMS2 and rest all (i.e.22 out of 31) had a score of more than 2.

### MMR expression in the core biopsy specimen of tumors before NACT

When MMR expression was rated as positive and negative, MLH1 and MSH2 staining was negative in around 40% of cases whereas MSH6 was negative in around 50% of cases, for PMS2 the negative expression was found in only 29.03% patients(tables 3, 4, 5, 6).

The H-score for MLH1 was better than that for MSH2, which showed poor staining in breast cancers. MSH6 was the most frequently altered MMR gene, with negativity rate of 48% while PMS2 was found to be the least altered MMR gene and it showed strong staining in most of the tumors(tables 3, 4, 5, 6)

There was no significant correlation observed between age, family history, T- size, lymph node status or histological sub-type of tumor and expression of MMR in the core cut biopsy. However, a **significant **correlation was observed between expression of MLH1 and MSH2 and histological grade (p = 0.048 and 0.038 respectively), tumors with negative or reduced H-scores of MLH1 and MSH2 had lesser degree of differentiation. Out of the twelve MSH2 negative cases in this study, four were poorly differentiated and only one was well differentiated, whereas, of the 19 MSH2 positive tumors 8 were well differentiated. Histological grade is a known predictor of poor outcome and this finding suggests that tumors with negative expression of MMR are likely to be more aggressive with a poor outcome in terms of survival.

The correlation of H-scores of all the four MMR gene products with objective clinical response was studied separately and tested for significance. No significant correlation was found between pre-NACT, H-scores and clinical response for MLH1, MSH2, and MSH6 however a statistically significant negative correlation was observed between PMS2 expression and clinical response (p = .045). Cases with high post NACT expression of PMS2 were poor responders of chemotherapy (CAF regimen).

### MMR expression after NACT

Immunohistochemistry was carried out after neoadjuvant chemotherapy in the mastectomy specimen and H-scores of all four MMR proteins were correlated with clinical response. It was found that although, after neo-adjuvant chemotherapy the mean H-scores of MLH1, MSH2 and MSH6 and PMS2 were reduced as compared to pre-NACT expression, the change in their expression was not statistically significant. However the mean H-score of PMS2 increased after chemotherapy, though the change was again not statistically significant. **There was no significant correlation observed between clinical response and H-scores of MLH1, MSH2, MSH6 and PMS2 after neoadjuvant chemotherapy **(Tables 6, 7, 8, 9, 10)

## Discussion

DNA mismatch repair (MMR) pathway is an important post-replicative repair process involved in the maintenance of genomic stability [5-9]. The MMR genes repair erroneous pairing of nucleotide bases, which can arise through DNA replication, genetic recombination, deamination of 5-methylcytosine to thymine and the action of chemical mutagens. Upon binding to mismatch, the complex protein undergoes a conformational change associated with exchange of bound ADP from ATP resulting in the formation of a clamp that can translocate along the DNA. Thus erroneous pairing is recognized, excised and replaced with the correct one[7,8].

The hallmark of MMR dysfunction is micro satellite instability (MSI).Micro satellites are repeated regions of one to six nucleotide units that occur primarily in non-coding regions and the number of micro satellite repeat units located at a given locus is genetically determined. Micro satellite instability can occur during replication of repetitive sequences when two strands of DNA-the strand that is being copied and the new one being synthesized, slip relative to one another resulting in small loops of unpaired DNA.

Normally, after replication, the mismatch repair system which is carried out by products of four mismatch repair genes (MMR) i.e. hMSH2, hMLH1, hPMS2 and hMSH6 and a few others, would recognize mismatched base pairs and replace them with correct nucleotides. The efficiency of heteroduplex repair is much reduced in cells with mutations in MMR genes [7-10].

These genes have been studied most extensively in colon cancers and have been found to impart certain clinical and pathological characteristics to the tumor [5-9]. In breast cancers also, MMR dysfunction has been studied quite extensively and in sporadic breast cancers the reported frequency of MSI has varied widely. Yee et al. [18] used seven micro satellites against twenty breast neoplasms and found instability in four of them (20%). At extreme odds with other reports Patel et al [19] detected MSI in 11 of 13 (85%) tumors using 9 microsattelites. The greatest problem in comparing the results of different series is the fact that each series generally used a different set of micro satellite markers thus making true comparison impossible [10-17].

In contrast to HNPCC syndrome there is less concordance between MSI and MMR gene dysfunctions in breast cancer. This absence of correlation between MMR function loss and MSI suggest oncogenetic mechanism of progression in primary breast cancer different from that in HNPCC. This lack of correlation between MMR dysfunction and MSI could be due to involvement of MMR genes not leading to MSI formation like MLH6 or PMS2.Thus in breast cancers immunohistochemistry for MMR protein products would appear to be more logical for detection of loss of MMR function [9-16].

The studies correlating the clinical pathological parameters of breast cancer with MMR dysfunction suggest an association between MSI and poor clinico- pathological features namely: larger tumor size, positive nodal status, advanced stage, negative expression of hormone receptors and decreased disease free and overall survival [9,12-14].

In the present study, no significant correlation was found between any of the MMR gene-products with age, family history, T-size, N-stage and histotype. There was a strong correlation observed between expression of MLH1 and MSH2 with the histological grading of tumor, which is a known predictor of poor outcome in breast cancer. The lack of correlation observed in the present study could be explained partly on the basis of selection criteria for this study, which included only locally advanced breast cancers.

MMR protein MutSα (a heterodimer of MSH2 and MSH6) recognizes and binds to sites of DNA damage, such as O^6^-methylguanine and 1,2-cisplatin intrastrand cross links, and is proposed to lead to recruitment of MutLα (a heterodimer of MLH1 and PMS2 O^6^-methylguanine) into the complex. This has been suggested to lead to either futile rounds of DNA repair or replication stalling and activation of an apoptotic pathway [14-17].

For topoisomerase II inhibitors (such as doxorubicin), MMR proteins may serve as a detector of the cleavable complex produced by the binding of the drug to topoisomerase II. Alternatively, doxorubicin is known to participate in redox cycling reactions that produce DNA damage including cross-links that may be recognized by MMR. Furthermore MMR activity can be restored to extracts of defective cell lines by complementation with complexes of hMSH2 and GTB, hMLH1 or PMS2 or by introducing a human chromosome with wild type copy of MLH1.However in most of cancers in a clinical scenario MSI and loss of MMR function is associated with a favorable response to chemotherapy. In breast cancers there have been few studies to delineate MMR dysfunction and response to chemotherapy [18-20].

The role of hMLH1 as predictor of response to therapy by correlation with disease-free survival was assessed in a study with patients receiving regimens containing doxorubicin or cisplatin, drugs exhibiting resistance in vitro associated with loss of MLH1 expression [12]. Further, the expression of MLH1 in tumors from women with clinically node-positive breast cancer was compared before and after primary (neoadjuvant) chemotherapy: Immunohistochemistry scores of MLH1 were made on 36 tru-cut prechemotherapy biopsies and 29 paired post chemotherapy tumor samples. MLH1 expression before chemotherapy did not predict disease-free survival or tumor response to chemotherapy. Low MLH1 expression after chemotherapy, however was an independent predictor of poor disease-free survival [12-21].

In another study Immunohistochemical studies were performed on 71 histological specimens of breast cancer taken from the patients treated with surgery and subsequent cyclophosphamide, methotrexate, and 5-fluorouracil (CMF) or cyclophosphamide, Adriamycin, and 5-fluorouracil (CAF) chemotherapy for stage II or III primary breast cancer[22]. Positive expression of hMLH1-IS and hMSH2-IS were 57.7% and 60.6%, respectively, and complete losses of hMLH1 and hMSH2 were observed in 4.2% of patients. Amongst the patients of advanced cancer with lymph node metastasis, those having a low hMLH1-IS had a significantly higher failure rate with the CMF regimen than those having a high hMLH1-IS (*p *= 0.03). No significant difference was noted in chemotherapeutic response according to hMLH1 and hMSH2 expression in the group receiving the CAF regimen[22].

In the present study no significant correlation was found between pre or post chemotherapy MLH1 or MSH2 expression or H-score and response. There was however a significant correlation between clinical response and H-score of PMS2 before neo-adjuvant chemotherapy using CAF regime (P = 0.045) while no significant correlation was found between post chemotherapy MLH1 expression and response.

Previous studies have linked MMR proteins to the activation of apoptosis through p53-dependent and p53-independent mechanisms. MMR-deficient cells exhibit variable defects in the induction of p53 and its related p73, which are activators of apoptosis. An interaction between PMS2, an MMR protein, and p73 has been described which results in stabilization of p73 and redistribution of PMS2 to the nuclear compartment. Exposure to cisplatin enhances the association between PMS2 and p73. Moreover, stimulation of the p73 pro-apoptotic function by cisplatin requires PMS2. These results suggest that PMS2 contributes to genome integrity not only through DNA repair but also by enhancing DNA damage-induced apoptosis [22-28].

No studies evaluating the predictive value of PMS2 in breast cancer chemotherapy has been done yet. In the present study an inverse relationship was found between H-score of PMS2 and response to CAF regimen chemotherapy. Tumors with low scores responded better to chemotherapy as seen in other tumors like colonic malignancies.

In the present study 54.83% of patients had a significant objective clinical response to NACT (in terms of reduction in tumor size and down staging of the axillary lymph node status). A significant negative correlation was observed between PMS2 H-score after neo-adjuvant chemotherapy and clinical response. The patients with high H-scores expression responded poorly to NACT. No studies evaluating the predictive value of PMS2 in breast cancer chemotherapy have been done yet and in the present study an inverse relationship was observed between H-score of PMS2 and response to CAF regimen chemotherapy.

There was no significant correlation observed between expression of MMR proteins and age of the patient, family history, tumor size or histological type of the tumor, while a strong negative correlation was observed between expression of MLH1 and MSH2 and histological grading of the tumor, which is a reliable predictors of outcome in the treatment of breast cancer. The study also highlights the possible role of MMR expression in predicting aggressive tumor behavior in view of the statistically significant negative correlation between MLH1 and MSH2 expression and histological grade. The tumors with low expression of MLH1 and MSH2 are expected to have a better outcome.

## Competing interests

The author(s) declare that they have no competing interests.

## Authors' contributions

CM designed and initiated the study and provided the surgical expertise, VB and DB contributed towards the statistical analysis and standaridizing the surgical procedures, BP collected the specimens, assisted in surgeries and also contributed towards designing of the study, AB and SS performed the histopathology and carried out the immunohistochemistry of the specimen.

All members read and approved the manuscript.
